# Safety and Effectiveness of Direct Oral Anticoagulants vs. Warfarin in Patients With Atrial Fibrillation and Endoscopy-Diagnosed Peptic Ulcer

**DOI:** 10.3389/fcvm.2021.774072

**Published:** 2021-12-23

**Authors:** Chun-Li Wang, Chien-Hao Huang, Victor Chien-Chia Wu, Ya-Chi Huang, Hsiang-Sheng Wang, Chang-Fu Kuo, Pao-Hsien Chu, Ming-Shien Wen, Ying-Jen Chen, Yu-Tung Huang, Shang-Hung Chang

**Affiliations:** ^1^Cardiovascular Division, Department of Internal Medicine, Linkou Medical Center, Chang Gung Memorial Hospital, Taoyuan, Taiwan; ^2^College of Medicine, Chang-Gung University, Taoyuan, Taiwan; ^3^Division of Gastroenterology and Hepatology, Department of Internal Medicine, Linkou Medical Center, Chang Gung Memorial Hospital, Taoyuan, Taiwan; ^4^Center for Big Data Analytics and Statistics, Linkou Medical Center, Chang Gung Memorial Hospital, Taoyuan, Taiwan; ^5^Department of Anatomic Pathology, Linkou Medical Center, Chang Gung Memorial Hospital, Taoyuan, Taiwan; ^6^Division of Rheumatology, Allergy and Immunology, Department of Internal Medicine, Linkou Medical Center, Chang Gung Memorial Hospital, Taoyuan, Taiwan; ^7^Division of General Internal Medicine and Geriatrics, Department of Internal Medicine, Linkou Medical Center, Chang Gung Memorial Hospital, Taoyuan, Taiwan; ^8^Graduate Institute of Nursing, Chang Gung University of Science and Technology, Taoyuan, Taiwan

**Keywords:** atrial fibrillation, direct oral anticoagulants, endoscopy, hemorrhage, peptic ulcer, safety

## Abstract

**Background:** Patients with active peptic ulcer (PU) were excluded from direct oral anticoagulant (DOAC) trials for stroke prevention in patients with atrial fibrillation (AF). This study evaluated the safety and effectiveness of DOACs in AF patients with active, inactive and no peptic ulcer (PU).

**Methods:** This study accessed electronic medical records from January 1, 2009 to May 31, 2019 at a multi-center healthcare provider in Taiwan and involved 2,955 AF patients who had undergone esophagogastroduodenoscopy ≤ 1 year before anticoagulation. Subjects were classified into 3 groups: active (*n* = 237), inactive (*n* = 828) and no-PU (*n* = 1,890) groups. We compared the risks of major bleeding, gastrointestinal bleeding, and ischemic stroke/systemic embolism (IS/SE) between DOACs and warfarin among the 3 groups.

**Results:** In the active PU group, there were no significant differences in the risks of major bleeding [hazard ratio (HR) = 0.65, 95% confidence interval (CI) 0.08–4.98, *p* = 0.676], gastrointestinal bleeding (HR = 0.65, 95% CI 0.08–4.98, *p* = 0.676) and IS/SE (HR = 2.58; 95% CI 0.53–12.70, *p* = 0.243) between DOAC and warfarin (as the reference). In the inactive PU group, there were no significant differences in the risks of major bleeding (HR = 0.36, 95% CI 0.09–1.39, *p* = 0.138), gastrointestinal bleeding (HR = 0.21, 95% CI 0.02–1.80, p = 0.153), and IS/SE (HR = 1.04, 95% CI 0.39–2.82, *p* = 0.934) between DOAC and warfarin (as the reference). In the no-PU group, DOACs were associated with lower risk of major bleeding (HR = 0.26, 95% CI 0.12–0.53, *p* < 0.001), gastrointestinal bleeding (HR = 0.25, 95% CI 0.01–0.59, *p* = 0.002), and similar risk of IS/SE (HR = 0.92, 95% CI 0.55–1.54, *p* = 0.757) compared to warfarin.

**Conclusions:** DOACs were as effective as warfarin in preventing IS/SE irrespective of PU status and safer than warfarin in reducing major bleeding in the no-PU group. In patients with active or inactive PUs, DOAC and warfarin were not significantly different in their effects on major bleeding or gastrointestinal bleeding.

## Introduction

Anticoagulation with warfarin or direct oral anticoagulants (DOACs) significantly reduces the risk of stroke in patients with atrial fibrillation (AF) (1, 2). The trade-off is an increased risk of gastrointestinal bleeding, especially in patients with a recent history of active peptic ulcer (PU) or gastrointestinal bleeding (1, 2). Gastrointestinal bleeding complicates long-term anticoagulation therapy in 5–15% of patients, and the gastrointestinal tract is the most common site of significant bleeding in patients receiving anticoagulant therapy (3). Among those with gastrointestinal bleeding, gastric and duodenal ulcers are the most common etiologies, accounting for approximately 45–70% of total cases (4, 5). A retrospective study in AF patients with a history of ulcer bleeding showed warfarin failed to improve outcomes, as the modest benefit in reducing major adverse cardiovascular events was offset by increased gastrointestinal bleeding (6).

DOACs have become increasingly preferred over warfarin, given their fewer food-drug or drug-drug interactions, rapid onset and offset, and lack of the need for frequent monitoring (7, 8). In AF patients without risk factors for major bleeding, DOACs are at least as effective as warfarin in preventing stroke and safer than warfarin in reducing major bleeding (8–10). However, patients with a recent history of active PU or gastrointestinal bleeding were mostly excluded from major DOAC trials for stroke prevention in AF (11–14). Furthermore, there is no specific recommendation on the use of anticoagulants in AF patients with a history of active PU or gastrointestinal bleeding in current guidelines (8, 15). Therefore, this study aimed to compare the safety and effectiveness of DOAC vs. warfarin for stroke prevention in patients with AF and endoscopic findings of active, inactive, and no PU.

## Methods

### Data Source

In this retrospective cohort study, patient data were collected from the electronic medical records of Chang Gung Memorial Hospital System, which is currently the largest healthcare provider in Taiwan, comprising three tertiary care medical centers and four major teaching hospitals. Among healthcare services reimbursed by the Taiwan's National Health Insurance, the Chang Gung Memorial Hospital system covered 21.2% outpatient services and 12.4% inpatient services (16). All patients' identification numbers were encrypted and de-identified to protect privacy. Laboratory data and diagnoses were linked and continuously monitored using consistent encryption. This study was performed during the period between May 01, 2018 and Apr 30, 2020. The institutional review board of Chang Gung Memorial Hospital approved the study protocol (approval serial number: 201900618B0) and has waived the need for informed consent.

### Study Population

**Figure 1:**
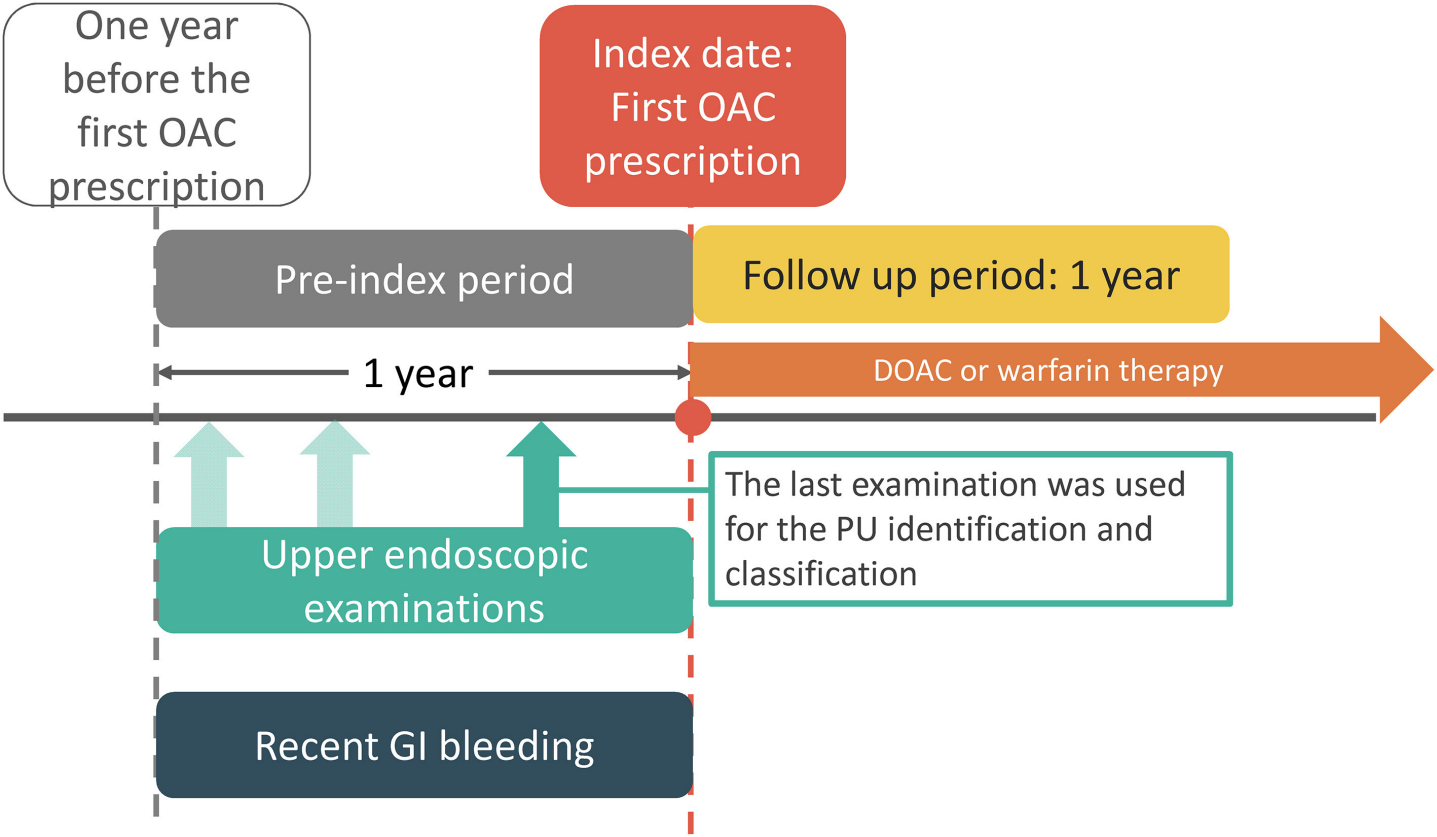
Study time frame. Index date is the date of the first OAC prescription. PU and recent GI bleeding events were identified from the pre-index 1 year period. DOAC, direct oral anticoagulant; GI, gastrointestinal; OAC, oral anticoagulant.

We included patients diagnosed with AF [identified via International Classification of Diseases, 9th Revision (ICD-9) code 427.31; or 10th Revision (ICD-10) codes I48.0, I48.1, I48.2, or I48.91] between January 1, 2009 and May 31, 2018 with at least one prescription filled for oral anticoagulants following the diagnosis. The oral anticoagulant included a DOAC (dabigatran, rivaroxaban, apixaban, or edoxaban) or warfarin. The index date was defined as the date of the first DOAC or warfarin prescription (Figure 1). Patients were excluded if they had met one of the following criteria: no upper endoscopic examination within 1 year before the index date, pulmonary embolism or deep vein thrombosis within 6 months prior to the index day, heart-valve replacement or joint surgery within 6 months prior to the index date, end-stage renal disease before the index date, ischemic stroke/systemic embolism (IS/SE), or death within 7 days after the index date, and concurrent use of DOAC and warfarin. Following exclusion, eligible patients were classified into three groups: active, inactive, and no PU groups according to the findings of upper endoscopic examinations within 1 year before the index date (Figure 1). Endoscopic grading of active and inactive PUs was categorized according to the Forrest classification (Supplementary Table 1). Active PU was defined as PUs that were actively bleeding (Forrest Ia, Ib) or had recently bled (Forrest IIa, IIb, IIc), while inactive PU was defined as clean base ulcers without signs of bleeding (Forrest III) (17). If there were more than one upper endoscopic examinations within 1 year before the index date, the last examination was chosen for the PU identification and classification. All selected subjects were followed up for 1 year.

### Other Covariates

Baseline comorbidities of the study cohort included hypertension, diabetes mellitus, chronic liver disease, chronic kidney disease, or past history of heart failure, myocardial infarction, IS/SE, bleeding, PU disease, or cancer. All these baseline comorbidities were defined by at least two outpatient clinic diagnosis or one inpatient discharge diagnosis using ICD-9 and ICD-10 codes (Supplementary Table 2). Laboratory data including estimated glomerular filtration rate, liver function tests, hemoglobin, and platelet counts, were calculated from values closest to the index date. Baseline medications were identified from medical records at the index date (±7 days). We used the CHA_2_DS_2_-VASc score [congestive heart failure (score of 1), hypertension (score of 1), age ≥75 years (score of 2), diabetes mellitus (score of 1), previous stroke or transient ischemic attack (score of 2), vascular disease (score of 1), age 65–74 years (score of 1), and female gender (score of 1)] to estimate the risk of ischemic stroke (18). We also used the HAS-BLED score (hypertension, abnormal renal or liver function, stroke, bleeding history, labile international normal ratio, age ≥65 years, and antiplatelet drug or alcohol use) to estimate the risk of major bleeding (19).

### Endpoints

The study endpoints included major bleeding, gastrointestinal bleeding, IS/SE, death, and composite of major bleeding, IS/SE, and death. Major bleeding was defined as clinically overt bleeding associated with at least a two g/dL decrease in hemoglobin, requiring a transfusion of at least two units of packed red blood cells or whole blood, fatal bleeding, or intracranial hemorrhage during the period of drug use or within the 14-day period after the last day of drug use. Gastrointestinal bleeding was defined as hospitalization with a primary diagnosis of bleeding in any segment of the gastrointestinal tract during the drug-use period or within 14 days after the last day of drug use. IS/SE was defined as admission with a primary discharge diagnosis of ischemic stroke or systemic thromboembolism. All ICD-9 and ICD-10 codes used to identify the endpoints are listed in Supplementary Table 2. Patients who changed their oral anticoagulants were censored 14 days following their switch in treatment. If an event or death occurred within 14 days following a switch, that event and time were ascribed to the initial therapy.

### Statistical Analysis

Data were presented as means ± standard deviation or as number (percentage) for categorical variables. Differences between continuous values were assessed using a student's *t*-test. Differences between categorical variables were compared with a χ^2^ test. We used a Cox proportional hazard regression analysis to estimate the risk of study outcomes between DOAC users and warfarin users in the three groups, expressed as hazard ratios (HR) with 95% confidence intervals (95% CI). The analysis was adjusted for covariates, including baseline characteristics, comorbidities, laboratory data, and medications. A *p*-value of < 0.05 was considered statistically significant. All analyses were performed using SAS software, version 9.4 (SAS Institute, Cary, North Carolina, USA).

### Sensitivity and Subgroup Analyses

We performed several sensitivity and subgroup analyses to validate our findings and identify potential biases. First, given the high mortality risk in AF patients with high CHA_2_DS_2_-VASc and HAS-BLED scores, we reanalyzed the data to account for competing risk of death. Second, we reclassified patients with PUs to those with and without recent gastrointestinal bleeding within 1 year and reanalyzed the data. We defined PU with recent gastrointestinal bleeding as PU with a primary admission diagnosis of gastrointestinal bleeding within 1 year before initiating anticoagulation (Figure 1). Third, a sensitivity analysis evaluated whether a change in the definition of active PU to Forrest Ia–IIb would affect the main result. Fourth, a sensitivity analysis evaluated whether a change in the study start date from January 1, 2009 to May 1, 2012 (the date of the first prescription of DOAC) would affect the main results. Fifth, we performed subgroup analyses for patients with and without non-steroidal anti-inflammatory drug use, as well as those with and without *Helicobacter pylori* infection based on histological examinations.

## Resutls

**Figure 2:**
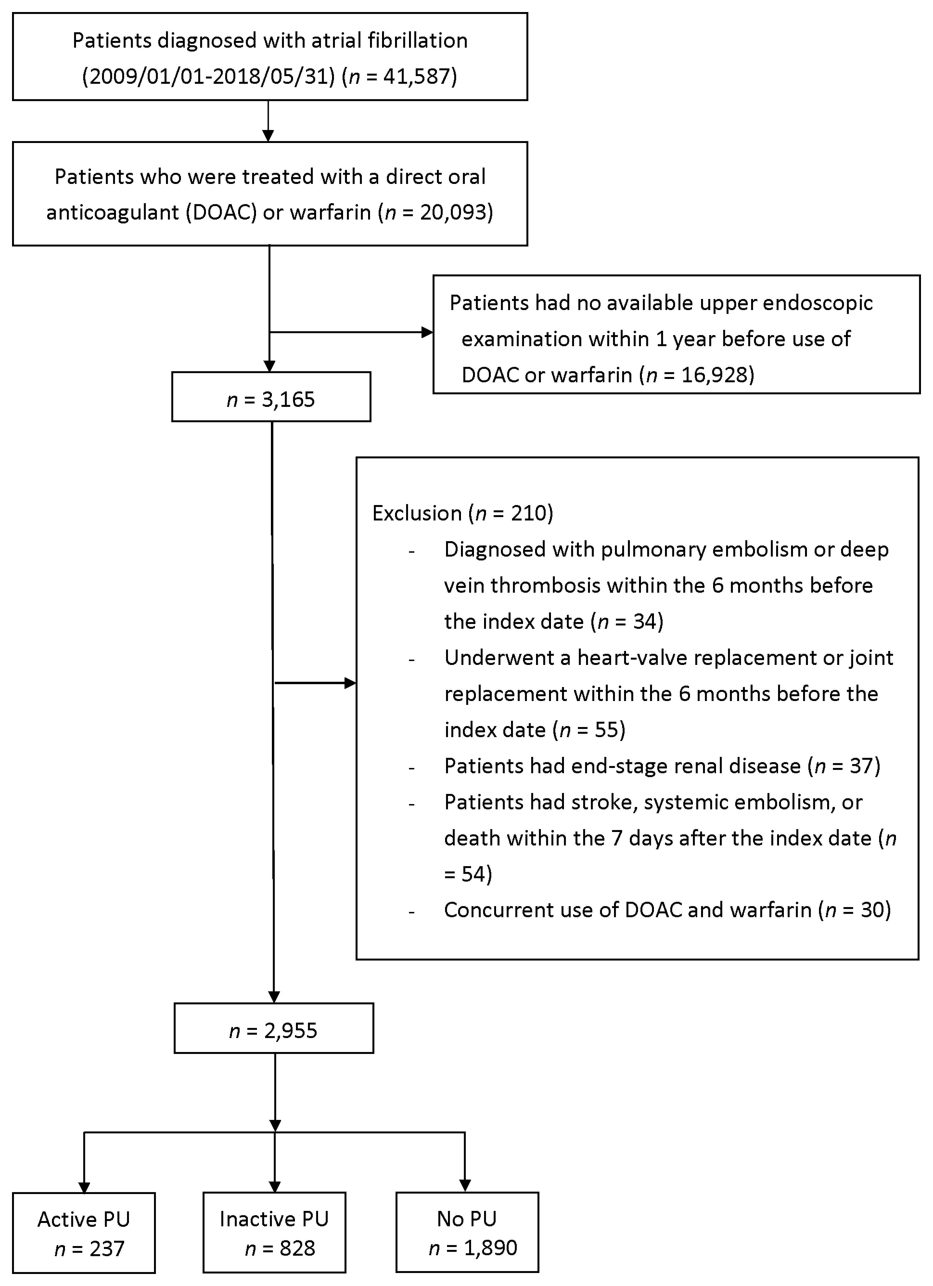
Enrollment of anticoagulated patients with AF who underwent esophagogastroduodenoscopy <1 year before initiating anticoagulation. AF, atrial fibrillation; PU, peptic ulcer.

During the period between January 1, 2009 and May 31, 2018, 20,093 patients were diagnosed with AF and received DOAC or warfarin therapy following the diagnosis (Figure 2). We excluded the following patients: 16,928 patients who had no upper endoscopic examination within 1 year before the index date, 34 patients who had pulmonary embolism or deep vein thrombosis within 6 months prior to the index day, 55 patients who had a heart-valve replacement or joint surgery within 6 months prior to the index date, 37 patients who had end-stage renal disease before the index date, 54 patients who had IS/SE or death within seven days after the index date, and 30 patients who had concurrent use of DOAC and warfarin. The reasons for an upper endoscopic examination included evaluating upper abdominal symptoms, persistent or recurrent esophageal reflux, dysphagia, odynophagia, persistent vomiting of unknown cause, or gastrointestinal bleeding (Supplementary Table 3).

### Participant Characteristics

Following exclusion, 2,955 patients remained eligible and were classified into three groups: active (*n* = 237, 8%), inactive (*n* = 828, 28%), and no PU (n = 1,890, 64%) groups. Patients in the active PU group were more likely to have a history of bleeding than inactive or no PU patients (Table 1). The HAS-BLED score was significantly higher in the active PU group, mainly due to a history of prior bleeding. The active PU group showed lower hemoglobin counts and more frequent use of proton pump inhibitors than the inactive and no PU groups. A separate comparison between DOAC and warfarin users in the active and inactive PU groups is shown in Supplementary Table 4. In the active PU group, DOAC users were older and had higher CHA_2_DS_2_-VASc and HAS-BLED scores than warfarin users. In the inactive PU group, DOAC users were older and had similar CHA_2_DS_2_-VASc and HAS-BLED scores compared to warfarin users.

**Table 1:** Patient characteristics at first OAC prescription.

	**Active PU (*n* = 237)**	**Inactive PU (*n* = 828)**	**No PU (*n* = 1,890)**	***p*-value**
**Demographic**
Age (years)	74.9 (9.9)^†^	73.1 (9.7)	72.4 (10.9)	0.003
Female	97 (40.9)	337 (40.7)	858 (45.4)	0.050
**Medical history**
Diabetes mellitus	95 (40.1)	343 (41.4)	783 (41.4)	0.922
Hypertension	188 (79.3)	633 (76.5)	1,428 (75.6)	0.423
Chronic liver disease	61 (25.7)	263 (31.8)	598 (31.6)	0.166
Heart failure	105 (44.3)	345 (41.7)	734 (38.8)	0.146
Myocardial infarction	57 (24.1)	187 (22.6)	366 (19.4)	0.065
Stroke	77 (32.5)	268 (32.4)	594 (31.4)	0.863
Bleeding history	188 (79.4)^*^,	461 (55.7)^†^	923 (48.8)	<0.001
Cancer	37 (15.6)	166 (20.1)	385 (20.4)	0.222
CHA_2_DS_2_-VASc score	4.3 ± 1.9^†^	4.1 ± 1.9	4.0 ± 1.9	0.047
HAS-BLED score	4.4 ± 1.3^*^,	4.2 ± 1.3^†^	4.0 ± 1.4	<0.001
Paroxysmal AF	81 (34.2)^*^	293 (35.4)	785 (41.5)	0.002
Body weight (kg)	63.6 ± 12.5	64.7 ± 12.8	63.6 ± 13.8	0.166
**Laboratory data**
eGFR (mL/min/1.73 m^2^)	62.6 ± 32.1	64.1 ± 35.0	67.1 ± 33.2	0.033
Hemoglobin (g/dL)	10.6 ± 1.9^*^,	11.7 ± 2.3^†^	12.1 ± 2.3	<0.001
Platelet (10^3^/uL)	218.0 ± 94.2^*^,	200.8 ± 74.6	201.5 ± 77.7	0.007
AST (u/L)	36.4 ± 46.8^*^,	30.9 ± 20.4	31.0 ± 22.9	0.025
ALT (u/L)	26.8 ± 25.7	27.0 ± 26.5	28.9 ± 35.0	0.301
Total bilirubin (mg/dL)	0.9 ± 0.7	0.9 ± 0.9	0.9 ± 0.6	0.812
LDL-C (mg/dL)	89.5 ± 26.6	93.2 ± 30.2	94.9 ± 30.6	0.345
**Medications^#^**
ß-blocker	118 (49.8)	429 (51.8)	962 (50.9)	0.835
RAAS inhibitor	124 (52.3)	421 (50.8)	965 (51.1)	0.921
Ca channel blocker	49 (20.7)	183 (22.1)	398 (21.1)	0.804
Loop diuretic	106 (44.7)	337 (40.7)	624 (33.0)	<0.001
Amiodarone	91 (38.4)	346 (41.8)	824 (43.6)	0.260
Digoxin	66 (27.9)	252 (30.4)	508 (26.9)	0.164
Statins	65 (27.9)	202 (24.4)	485 (25.7)	0.601
Aspirin	36 (15.2)	157 (19.0)	347 (18.4)	0.411
Clopidogrel	85 (35.9)^†^	227 (27.4)^†^	388 (20.5)	<0.001
NSAID	19 (8.0)	64 (7.7)	156 (8.3)	0.898
PPI	112 (47.3)^†^	331 (40.0)^†^	485 (25.7)	<0.001
Steroid	24 (10.1)	84 (10.1)	136 (7.2)	0.020
Warfarin	121 (51.1)	390 (47.1)	878 (46.5)	0.408
**DOAC**
Dabigatran 110 mg	39 (16.5)	116 (14.0)	323 (17.1)	0.132
Dabigatran 150 mg	7 (3.0)	28 (3.4)	59 (3.1)	0.919
Rivaroxaban 10 mg	33 (13.9)	100 (12.1)	228 (12.1)	0.705
Rivaroxaban 15 mg	37 (15.6)	155 (18.7)	398 (21.1)	0.081
Rivaroxaban 20 mg	23 (9.7)	82 (9.9)	191 (10.1)	0.973
Apixaban 5 mg	38 (16.0)	187 (22.6)^†^	330 (17.5)	0.004
Edoxaban 30 mg	25 (10.6)	64 (7.7)	168 (8.9)	0.352
Edoxaban 60 mg	8 (3.4)	30 (3.6)	58 (3.1)	0.750

*Data are presented as mean ± standard deviation or n (%)*.

*DOAC, direct oral anticoagulant; eGFR, estimated glomerular filtration rate; NSAID, non-steroidal anti-inflammatory drug; OAC, oral anticoagulant; PPI, proton pump inhibitor; RAAS, renin–angiotensin–aldosterone system*.

* *p < 0.05 vs. inactive PU group*.

† *p < 0.05 vs. no PU group*.

# *Medications were identified from medical records at the index date (±7 days)*.

### Patients With Active PU

**Figure 3:**
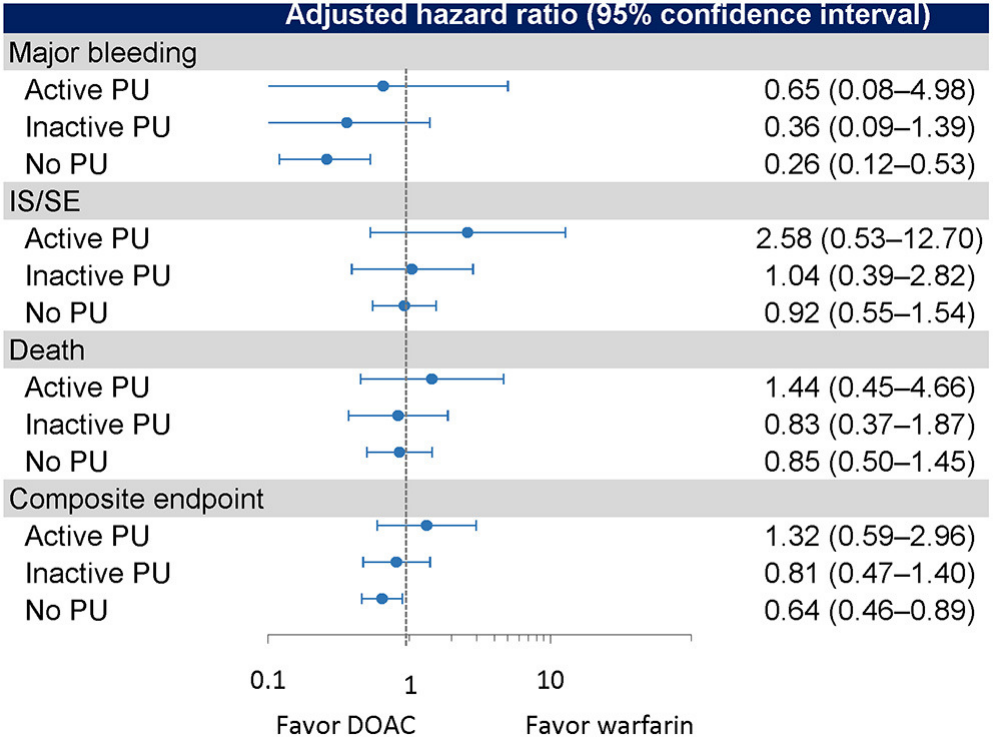
Event rates and risks of outcomes in the active PU, inactive PU, and no PU groups. PU, peptic ulcer.

The crude event rates per 100 person-years in DOAC (*n* = 124) and warfarin (*n* = 113) users were 2.35 and 4.04 for major bleeding, 2.35 and 4.04 for gastrointestinal bleeding, 9.64 and 2.70 for IS/SE, 8.22 and 7.92 for death, and 18.09 and 15.17 for composite endpoint, respectively (Table 2). There were no significant differences in the risks of IS/SE (adjusted HR = 2.58, 95% CI 0.53–12.70, *p* = 0.243), major bleeding (adjusted HR = 0.65, 95% CI 0.08–4.98, *p* = 0.676), gastrointestinal bleeding (adjusted HR = 0.65, 95% CI 0.08–4.98, *p* = 0.676), death (adjusted HR = 1.44, 95% CI 0.45–4.66, *p* = 0.538) and composite endpoint (adjusted HR = 1.32, 95% CI 0.59–2.96, *p* = 0.507) between DOAC and warfarin (as the reference) (Figure 3).

**Table 2:** Event rate and risk of major bleeding, GI bleeding, IS/SE, death, and composite endpoint in anticoagulated patients with AF, stratified by endoscopic findings of PU status.

	**Events**, ***n*** **(%)**		**Event rate /100 person-years**		**Crude HR (95% CI)**	***p*-value**	Adjusted HR^*^ (95% CI)	***p*-value**	Competing risk^†^ HR^*^ (95% CI)	***p*-value**
	**DOACs**	**Warfarin**	**DOACs**	**Warfarin**						
Active PU	*(n* = 124)	*(n* = 113)								
Major bleeding	2 (1.61%)	3 (2.65%)	2.35	4.04	0.58 (0.10–3.45)	0.546	0.65 (0.08–4.98)	0.676	0.66 (0.12–3.51)	0.625
GI bleeding	2 (1.61%)	3 (2.65%)	2.35	4.04	0.58 (0.10-3.45)	0.546	0.65 (0.08–4.98)	0.676	0.66 (0.12–3.51)	0.625
IS/SE	8 (6.45%)	2 (1.77%)	9.64	2.70	3.54 (0.75–16.66)	0.110	2.58 (0.53–12.70)	0.243	2.71 (0.37–19.74)	0.327
Death	7 (5.65%)	6 (5.31%)	8.22	7.92	1.03 (0.35–3.07)	0.957	1.44 (0.45–4.66)	0.538		
Composite	15 (12.10%)	11 (9.73%)	18.09	15.17	1.19 (0.55–2.59)	0.662	1.32 (0.59–2.96)	0.507		
Inactive PU	*(n* = 451)	*(n* = 377)								
Major bleeding	3 (0.67%)	11 (2.92%)	0.93	3.89	0.24 (0.07–0.86)	0.028	0.36 (0.09–1.39)	0.138	0.36 (0.10–1.32)	0.124
GI bleeding	1 (0.22%)	8 (2.12%)	0.31	2.82	0.11 (0.01–0.89)	0.038	0.21 (0.02–1.80)	0.153	0.21 (0.03–1.77)	0.151
IS/SE	9 (2.00%)	9 (2.39%)	2.83	3.19	0.86 (0.34–2.18)	0.756	1.04 (0.39–2.82)	0.934	1.04 (0.41–2.68)	0.929
Death	15 (3.33%)	12 (3.18%)	4.66	4.18	1.07 (0.50–2.28)	0.865	0.83 (0.37–1.87)	0.657		
Composite	27 (5.99%)	31 (8.22%)	8.49	11.18	0.75 (0.45–1.25)	0.264	0.81 (0.47–1.40)	0.450		
No PU	*(n* = 1,053)	*(n* = 837)								
Major bleeding	10 (0.95%)	35 (4.18%)	1.40	5.61	0.24 (0.12–0.49)	<0.001	0.26 (0.12–0.53)	<0.001	0.26 (0.13-0.51)	<0.001
GI bleeding	7 (0.66%)	25 (2.99%)	0.98	3.99	0.24 (0.01–0.55)	0.001	0.25 (0.01–0.59)	0.002	0.25 (0.01-0.57)	0.001
IS/SE	33 (3.13%)	31 (3.70%)	4.67	4.98	0.92 (0.56–1.50)	0.726	0.92 (0.55–1.54)	0.757	0.91 (0.54-1.55)	0.725
Death	33 (3.13%)	26 (3.11%)	4.59	4.08	1.08 (0.65–1.81)	0.766	0.85 (0.50–1.45)	0.558		
Composite	73 (6.93%)	84 (10.04%)	10.37	13.75	0.73 (0.54–1.00)	0.053	0.64 (0.46–0.89)	0.007		

*AF, atrial fibrillation; CI, confidence interval; DOAC, direct oral anticoagulant; GI, gastrointestinal; HR, hazard ratio; IS/SE, ischemic stroke/systemic embolism; PU, peptic ulcer*.

*Active and inactive PU were defined as Forrest Ia-IIc and Forrest III ulcer lesions according to the Forrest classification, respectively*.

* *Major bleeding adjusted for age, sex, hypertension, diabetes mellitus, chronic liver disease, history of heart failure, estimated glomerular filtration rate <60 mL/min/1.73 m^2^, cancer, history of peptic ulcer disease, history of bleeding, history of stroke, non-steroidal anti-inflammatory drug, proton pump inhibitors, and anti-platelets. IS/SE or death or composite, adjusted for age, sex, hypertension, diabetes mellitus, heart failure, estimated glomerular filtration rate <60 mL/min/1.73 m^2^, cancer, prior stroke, prior myocardial infarction, and statins*.

† *Death was considered as a competing risk factor in the Cox model*.

### Patients With Inactive PU

The crude event rates per 100 person-years in DOAC (*n* = 451) and warfarin (*n* = 377) users were 0.93 and 3.89 for major bleeding, 0.31 and 2.82 for gastrointestinal bleeding, 2.83 and 3.19 for IS/SE, 4.66 and 4.18 for death, and 8.49 and 11.18 for composite endpoint, respectively (Table 2). There were no significant differences in the risks of major bleeding (adjusted HR = 0.36, 95% CI 0.09–1.39, *p* = 0.138), gastrointestinal bleeding (adjusted HR = 0.21, 95% CI 0.02–1.80, *p* = 0.153), IS/SE (adjusted HR = 1.04, 95% CI 0.39–2.82, *p* = 0.934), death (adjusted HR = 0.83, 95% CI 0.37–1.87, *p* = 0.545), and composite endpoint (adjusted HR = 0.81, 95% CI 0.47–1.40, p = 0.450) between DOAC and warfarin (as the reference) (Figure 3).

### Patients Without PU

The crude event rates per 100 person-years in DOAC (*n* = 1,053) and warfarin (*n* = 837) users were 1.40 and 5.61 for major bleeding, 0.98 and 3.99 for gastrointestinal bleeding, 4.67 and 4.98 for IS/SE, 4.59 and 4.08 for death, and 10.37 and 13.75 for composite endpoint, respectively (Table 2). DOACs were associated with significantly lower risks of major bleeding (adjusted HR = 0.26, 95% CI 0.12–0.53, *p* < 0.001), gastrointestinal bleeding (adjusted HR = 0.25, 95% CI 0.01–0.59, *p* = 0.002), and composite endpoint (adjusted HR = 0.64, 95% CI 0.46–0.89, *p* = 0.007). No significant differences in the risks of IS/SE (adjusted HR = 0.92, 95% CI 0.55–1.54, *p* = 0.757) and death (adjusted HR = 0.85, 95% CI 0.50–1.45, *p* = 0.558) were observed between DOAC and warfarin (as the reference) (Figure 3).

### Sensitivity and Subgroup Analyses

The findings were similar in terms of major bleeding and IS/SE when death was viewed as a competing event in the Cox model (Table 2). The results were consistent with the main results when PU patients were reclassified according to gastrointestinal bleeding within 1 year before initiating anticoagulation (Table 3). We reanalyzed the data using Forrest Ia–IIb ulcer lesions to define PU with and without high bleeding risk and found similar results to those obtained in the main results (Supplementary Table 5). The results were consistent with the main results when the study start date changed from January 1, 2009 to May 1, 2012 (Supplementary Table 6). In the subgroup analyses, we found a statistically insignificant lower risk of major bleeding associated with DOAC vs. warfarin therapies in patients without non-steroidal anti-inflammatory drug use (adjusted HR = 0.21, 95% CI 0.04–1.01, *p* = 0.051; Supplementary Table 7). In patients showing *Helicobacter pylori* infection (*n* = 90), we observed a lower event rate of major bleeding in DOAC users than warfarin users (Supplementary Table 8).

**Table 3:** Event rate and risk of major bleeding, IS/SE, death, and composite endpoint in anticoagulated patients with AF, stratified by PU with and without recent GI bleeding.

	**Events**, ***n*** **(%)**		**Event rate /100 person-years**		**Crude HR (95% CI)**	***p*-value**	Adjusted HR^*^ (95% CI)	***p*-value**
	**DOACs**	**Warfarin**	**DOACs**	**Warfarin**				
PU with recent GI bleeding^†^	*(n* = 155)	*(n* = 169)						
major bleeding	2 (1.29%)	3 (1.78%)	1.83	2.64	0.69 (0.12–4.12)	0.683	0.62 (0.08–4.69)	0.645
IS/SE	9 (5.81%)	4 (2.37%)	8.44	3.56	2.37 (0.73–7.69)	0.152	2.02 (0.60–6.76)	0.255
Death	8 (5.16%)	7 (4.14%)	7.31	6.07	1.21 (0.44–3.33)	0.717	1.53 (0.52–4.53)	0.440
Composite	17 (10.97%)	14 (8.28%)	15.95	12.62	1.27 (0.62–2.57)	0.515	1.37 (0.66–2.84)	0.407
PU without recent GI bleeding^†^	*(n* = 420)	*(n* = 321)						
major bleeding	3 (0.71%)	11 (3.43%)	1.01	4.53	0.22 (0.06–0.80)	0.021	0.36 (0.09–1.43)	0.146
IS/SE	8 (1.90%)	7 (2.18%)	2.72	2.87	0.91 (0.33–2.52)	0.862	1.32 (0.45–3.92)	0.616
Death	14 (3.33%)	11 (3.43%)	4.71	4.44	1.00 (0.45–2.20)	0.993	0.74 (0.32–1.71)	0.475
Composite	25 (5.95%)	28 (8.72%)	8.49	11.73	0.70 (0.41–1.21)	0.202	0.78 (0.44–1.38)	0.385

*AF, atrial fibrillation; CI, confidence interval; DOACs, direct oral anticoagulants; GI, gastrointestinal; HR, hazard ratio; IS/SE, ischemic stroke/systemic embolism; PU, peptic ulcer*.

* *Major bleeding, adjusted for age, sex, hypertension, diabetes mellitus, chronic liver disease, heart failure, estimated glomerular filtration rate <60 mL/min/1.73m^2^, bleeding history, cancer, non-steroidal anti-inflammatory drug, proton pump inhibitors, and anti-platelets, and steroid. IS/SE, death or composite, adjusted for age, sex, hypertension, diabetes mellitus, heart failure, estimated glomerular filtration rate <60 mL/min/1.73 m^2^, cancer, prior stroke, prior myocardial infarction, and statins*.

† *Recent GI bleeding was identified as patients with a primary admission diagnosis of GI bleeding within 1 year before initiating anticoagulation*.

## Discussion

This is the largest study on real-world safety and effectiveness data for DOACs vs. warfarin in patients with AF and different endoscopic stages of PU reported to date. In the active and inactive PU groups, DOACs were associated with similar risks of IS/SE, major bleeding, and death compared to warfarin. In the no PU group, DOACs were associated with a significantly lower risk of major bleeding and similar risks of IS/SE and death compared to warfarin. Similar results were found when PU patients were reclassified according to gastrointestinal bleeding within 1 year before initiating anticoagulation.

Our study shows outcomes of DOAC therapy vary with pre-anticoagulation endoscopic findings. The advantage of DOAC vs. warfarin regarding major bleeding was found in the no PU group, but not in patients with PUs (either active or inactive). In patients with active PUs, DOAC did not appear to outperform warfarin in terms of decreasing major bleeding, and had similar efficacy in preventing IS/SE. Since no prior studies have compared DOACs with warfarin in AF patients with endoscopy-diagnosed PUs, our findings are novel and present new insights for managing these patients. Our study findings may convince clinicians to consider screening for PU status prior to initiating anticoagulation. In AF patients without PU, DOACs are generally favored over warfarin due to their efficacy and lower risk of major bleeding. In AF patients with a recent history of endoscopy-diagnosed PU or gastrointestinal bleeding, DOAC did not outperform warfarin in reducing major bleeding. Decision to prescribe an anticoagulant must be carefully weighed in such patients.

AF patients with a recent history of PU require more close follow-ups while receiving anticoagulants due to high risks of gastrointestinal bleeding and anticoagulation interruption.

Few previous studies have evaluated the impact of endoscopic findings on outcomes in patients receiving anticoagulant therapy (4, 9, 20, 21). Most studies were performed in scenarios with acute gastrointestinal bleeding and in patients who had received warfarin and not DOAC therapy. In a cohort study of 111 hospitalized patients with acute gastrointestinal bleeding while on warfarin, patients with no identifiable cause of bleeding or minimal upper endoscopic lesions showed more favorable outcomes and lower rates of re-bleeding and emergency surgery than those with high-risk endoscopic lesions (4). In a study of 1,329 warfarin-treated AF patients with gastrointestinal bleeding, warfarin resumption was associated with a higher risk of recurrent bleeding in patients with an active PU compared with non-resumption (21). Our findings corroborate these results, showing active PU lesions (Forrest Ia–IIc) are associated with high rates of major bleeding, IS/SE, and death in DOAC users. Our safety finding differs from a study on anemic AF patients receiving anticoagulation (9). In anemic patients with a history of PU (*n* = 220), DOAC therapy was associated with a significantly lower risk of major bleeding (HR = 0.36, 95% CI 0.21–0.61) than warfarin therapy (9). In that study, PU disease was identified from ICD codes, not from upper endoscopy, and PU history was not limited to 1 year (9).

DOAC has emerged as a leading oral anticoagulant that provides both patients and physicians with more effective, safe, and convenient therapeutic options in preventing thromboembolism. Before initiating a DOAC in patients with a recent history of PU, their history of major bleeding and clinical conditions likely to increase risk of bleeding, e.g., unhealed ulcer lesions, non-steroidal anti-inflammatory drug use, anemia, thrombocytopenia, impaired renal or liver function, should be evaluated, and corrected, if reversible (9, 10, 22). Since the rapid onset of anticoagulation effects and the short half-life of DOACs make initiating and interrupting therapy easier than with warfarin, DOACs should be prescribed as soon as feasible after establishing hemostasis. It is also crucial to avoid under- or over-dose prescriptions and to be consistent with labeled DOAC dose when treating patients who are at high risks of re-bleeding and ischemic stroke.

This study has some limitations. First, because it was a retrospective data analysis rather than a randomized controlled trial, it has inherent limitations, such as selection bias, reporting bias, and unmeasured confounding, despite statistical adjustments. Second, we did not assess the quality of anticoagulation control in the warfarin group as represented by the time in therapeutic range, which needs careful interpretation for the warfarin comparator. Third, our results reflected situations in the uniform ethnicity in Taiwan. Future studies are required to generalize the findings to other populations. Fourth, our analyses were based on any prescription filled and did not take into account dose or treatment duration. Fifth, it is possible that patients with healing ulcers would have been included in the active PU group given their initial findings of active PU. Sixth, the study cohort may be not optimally treated as <30% of patients were taking a statin despite the high proportion of patients with previous stroke, heart failure, or diabetes mellitus. This may influence the outcomes especially IS/SE and mortality (23). Finally, our observations about insignificant lower risk of major bleeding in the inactive PU group for DOAC users are probably due to limited sample size. It is possible that DOACs are safer than warfarin in patients with an inactive PU if the sample size is large enough. Further research might be warranted to confirm this finding.

## Conclusions

DOACs were as effective as warfarin in preventing IS/SE irrespective of PU status and safer than warfarin in reducing major bleeding in AF patients with endoscopic findings of no PU. In patients with active or inactive PUs, DOACs and warfarin were not significantly different in their effects on major bleeding. Our study findings may convince clinicians to consider screening for PU status prior to initiating anticoagulation in patients with AF.

## Data Availability Statement

The original contributions presented in the study are included in the article/Supplementary Material, further inquiries can be directed to the corresponding author/s.

## Ethics Statement

The studies involving human participants were reviewed and approved by Chang Gung Medical Foundation Institutional Review Board. Written informed consent for participation was not required for this study in accordance with the national legislation and the institutional requirements.

## Author Contributions

C-LW, C-HH, VW, Y-TH, Y-CH, and S-HC contributed to the design of the study. Y-TH and Y-CH were responsible for statistical analysis. C-LW and S-HC were responsible for drafting of the manuscript. All authors critically revised the manuscript for important intellectual content. All authors have approved the final version of this manuscript.

## Funding

This work was funded by research grants from the Chang Gung Memorial Hospital, Linkou, Taoyuan, Taiwan (CMRPG 3I0093).

## Conflict of Interest

The authors declare that the research was conducted in the absence of any commercial or financial relationships that could be construed as a potential conflict of interest.

## Publisher's Note

All claims expressed in this article are solely those of the authors and do not necessarily represent those of their affiliated organizations, or those of the publisher, the editors and the reviewers. Any product that may be evaluated in this article, or claim that may be made by its manufacturer, is not guaranteed or endorsed by the publisher.
