# Supplementary material for: Safety and Effectiveness of Direct Oral Anticoagulants vs. Warfarin in Patients With Atrial Fibrillation and Endoscopy-Diagnosed Peptic Ulcer
Source: Front Cardiovasc Med. 2021 Dec 23;8:774072. doi: 10.3389/fcvm.2021.774072 (PMC8732988; doi:10.3389/fcvm.2021.774072)
Supplement: Supplementary file 1 [file Data_Sheet_1.docx]

**SUPPLEMENTARY MATERIALS**

**Supplemental Table 1** Page 2

Classification of active and inactive peptic ulcer according to the Forrest classification

**Supplemental Table 2** Pages 3-4

ICD-9-CM and ICD-10-CM codes used to define the comorbidities and outcomes

**Supplemental Table 3** Page 5

Indications for upper endoscopy

**Supplemental Table 4** Pages 6-7

Baseline characteristics of DOACs and warfarin users in patients with active and inactive peptic ulcer

**Supplemental Table 5** Page 8

Sensitivity analysis: DOAC versus warfarin in patients with PU with and without high bleeding risk

**Supplemental Table 6** Pages 9-10

Sensitivity analysis: the study start date changed from January 1, 2009 to May 1, 2012

**Supplemental Table 7** Page 11

Rates and risk of outcomes in patients with peptic ulcer and NSAID use

**Supplemental Table 8** Page 12

Event rate and risk of outcomes in AF patients with and without Helicobacter Pylori infection detected by histological examinations

**Supplemental Table 1** Classification of active and inactive peptic ulcer according to the Forrest classification

| **Active peptic ulcer (*n* = 237)** | |  | **Inactive peptic ulcer (*n* = 828)** |
| --- | --- | --- | --- |
| Forrest I ulcer (*n* = 80):  active hemorrhage | Forrest II ulcer (*n* = 157):  signs of recent hemorrhage |  | Forrest III ulcer: lesions without active hemorrhage (*n* = 828) |
| - Ia: spurting hemorrhage | - IIa: non-bleeding visible vessel |  | - Clean-based ulcer |
| - Ib: oozing hemorrhage | - IIb: adherent clot |  |  |
|  | - IIc: hematin-covered lesion |  |  |

**Supplemental Table 2** ICD-9-CM and ICD-10-CM codes used to define the comorbidities and outcomes

| **Comorbidities and outcomes** | **ICD-9 codes** | **ICD-10 codes** | **Diagnosis definition** |
| --- | --- | --- | --- |
| End stage renal disease | 585 | N185, N186 | Discharge |
| Pulmonary embolism | 415.1, 415.11, 415.19 | I26 | Discharge |
| Deep vein thrombosis | 451.11, 451.19, 451.2, 451.81, 451.9, 453.40, 453.41,453.42,453.8, 453.9 | I82.4, I82.5, I82.6, I82.7 | Discharge |
| Total knee replacement, total hip replacement | 81.51, 81.54 | Z96.64, Z96.65 | Discharge |
| Heart valve replacement | 68016, 68017, 68018 |  | Discharge |
| Ischemic stroke | 433, 434, 435, 436 | I63, I64 | Discharge |
| Systemic embolism | 444 | I74 | Discharge |
| Intracranial hemorrhage | 430, 431, 432, 852, 853 | I60, I61, I62 | Discharge |
| Gastrointestinal bleeding | 456.0, 455.2, 455.5, 455.8, 530.7, 530.82, 531.0, 531.2, 531.4, 531.6, 532.0, 532.2, 532.4, 532.6, 533.0, 533.2, 533.4, 533.6, 534.0, 534.2, 534.4, 534.6, 569.3, 535.01, 535.11, 535.21, 535.31, 535,41, 535.51, 535.61, 535.71, 537.83, 537.84, 562.02, 562.03, 562.12 562.13, 569.3, 569.85, 578 | K250, K260, K270, K280 | Discharge |
| Other critical site bleeding | 423,0, 459.0, 568.81, 593.81, 599.7, 623.8, 626.32, 626.6, 719.1, 784.7, 784.8, 786.3 | D62, J942, H113, H356, H431, N02, N95, R04, R31, R58 | Discharge |
| Congestive heart failure | 428 | I11.0, I13.0, I13.2, I42.0,I50, I50.1, I50.91 | Discharge |
| Hypertension | 401, 402 | I10, I11 | Outpatient department ≥2 |
| Diabetes mellitus | 250 | E10, E10.1, E10.9, E11.0, E11.1, E11.9 | Outpatient department ≥2 |
| Chronic lung disease | 490, 491.0, 491.1, 491.20-491.22, 491.8, 491.9, 492.0, 492.8, 493.00-493.02, 493.10-493.12, 493.20-493.22, 493.81, 493.82, 493.90-493.92,494.0, 494.1, 495.8, 495.9, 496, 500, 502, 503, 504, 505, A323, A325 | J44 | Discharge |
| Chronic liver disease | 570, 571, 572 | B150, B160, B162, B190, K704, K72, K766, I85 | Outpatient department ≥2 |
| Chronic kidney disease | 580-589 | I12, I13, N00, N01, N02, N03, N05, N07, N11, N14, N17, N18, N19,Q61 | Outpatient department ≥2 |
| Coronary artery disease | 414.0, 414.2, 414.3, 414.4, 414.8, 414.9 | I25.1, I25.2, I25.4, I25.5, I25.6, I25.7, I25.8, I25.9 | Discharge |
| Myocardial infarction | 410, 411, 412 | I21-I25 | Discharge |
| Peripheral artery occlusive disease | 440.2 | I70.2-I70.9, I71, I73.9 | Discharge |

*ICD-9, International Classification of Diseases, Ninth Revision; ICD-10, International Classification of Diseases, Tenth Revision.*

**Supplemental Table 3.** Indications for upper endoscopy

|  | **Active PU** | | **Inactive PU** | | **No PU** | |
| --- | --- | --- | --- | --- | --- | --- |
| **Indications** | **DOAC**  **(*n* = 124)** | **Warfarin**  **(*n* = 113)** | **DOAC**  **(*n* = 451)** | **Warfarin**  **(*n* = 377)** | **DOAC**  **(*n* = 1,053)** | **Warfarin**  **(*n* = 837)** |
| Abdominal symptom or sign | 34 (27.4%) | 23 (20.4%) | 114 (25.3%) | 112 (29.7%) | 295 (28.0%) | 232 (27.7%) |
| Esophageal reflux | 30 (24.2%) | 33 (29.2%) | 135 (29.9%) | 123 (32.3%) | 424 (40.3%) | 327 (39.1%) |
| Dysphagia or odynophagia | 5 (4.0%) | 1 (0.9%) | 17 (3.8%) | 12 (3.2%) | 38 (3.6%) | 25 (3.0%) |
| Vomitting | 1 (0.8%) | 1 (0.9%) | 2 (0.4%) | 4 (1.1%) | 12 (1.1%) | 14 (1.7%) |
| Gastrointestinal bleeding | 21 (16.9%) | 29 (25.7%) | 46 (10.2%) | 37 (9.8%) | 72 (6.8%) | 40 (4.8%) |
| Others | 33 (26.6%) | 26 (23.0%) | 137 (30.3%) | 89 (23.6%) | 212 (20.1%) | 199 (23.8%) |

*DOAC, direct oral anticoagulant; PU*, peptic ulcer.

**Supplemental Table 4** Baseline characteristics of DOACs and warfarin users in patients with active and inactive peptic ulcer

|  | **Active peptic ulcer** | |  |  | **Inactive peptic ulcer** | |  |
| --- | --- | --- | --- | --- | --- | --- | --- |
| **Variable** | **DOACs**  **(*n* = 124)** | **Warfarin**  **(*n* = 113)** | ***p*-value** |  | **DOACs**  **(*n* = 451)** | **Warfarin**  **(*n* = 377)** | ***p*-value** |
| Demographic |  |  |  |  |  |  |  |
| Age (years) | 77.1 ± 8.5 | 72.4 ± 10.8 | <0.001 |  | 74.9 ± 9.0 | 70.9 ± 10.1 | <0.001 |
| Female | 50 (40.3) | 47 (41.6) | 0.843 |  | 179 (39.7) | 158 (41.9) | 0.517 |
| Medical history |  |  |  |  |  |  |  |
| Diabetes mellitus | 52 (41.9) | 43 (38.1) | 0.542 |  | 193 (42.8) | 150 (39.8) | 0.382 |
| Hypertension | 106 (85.5) | 82 (72.6) | 0.014 |  | 355 (78.7) | 278 (73.7) | 0.093 |
| Chronic liver disease | 31 (25.0) | 30 (26.6) | 0.785 |  | 139 (30.8) | 124 (32.89) | 0.524 |
| Heart failure | 48 (38.7) | 57 (50.4) | 0.069 |  | 156 (34.6) | 189 (50.1) | <0.001 |
| Myocardial infarction | 30 (24.2) | 27 (23.9) | 0.957 |  | 106 (23.5) | 81 (21.5) | 0.489 |
| Stroke | 43 (34.7) | 34 (30.1) | 0.451 |  | 139 (30.8) | 129 (34.2) | 0.298 |
| Bleeding history | 97 (78.2) | 91 (80.5) | 0.662 |  | 244 (54.1) | 217 (57.6) | 0.319 |
| Cancer | 16 (12.9) | 21 (18.6) | 0.229 |  | 97 (21.5) | 69 (18.3) | 0.251 |
| CHA_2_DS_2_-VASc score* | 4.6 ± 1.7 | 4.1 ± 2.0 | 0.030 |  | 4.2 ± 1.8 | 4.0 ± 1.9 | 0.137 |
| HAS-BLED score^†^ | 4.6 ± 1.2 | 4.3 ± 1.4 | 0.031 |  | 4.2 ± 1.3 | 4.1 ± 1.3 | 0.282 |
| Laboratory data |  |  |  |  |  |  |  |
| eGFR (mL/min/1.73m^2^) | 64.9 ± 25.9 | 60.1 ± 37.8 | 0.262 |  | 68.0 ± 28.8 | 59.4 ± 40.7 | <0.001 |
| Hemoglobin (g/dL) | 10.6 ± 1.9 | 10.6 ± 1.9 | 0.802 |  | 11.9 ± 2.3 | 11.5 ± 2.3 | 0.021 |
| Platelet (10^3^/uL) | 210.9 ± 73.5 | 225.8 ± 112.5 | 0.234 |  | 206.8 ± 74.9 | 193.6 ± 73.7 | 0.011 |
| AST (u/L) | 36.9 ± 42.8 | 35.7 ± 51.9 | 0.874 |  | 30.7 ± 22.8 | 31.1 ± 17.0 | 0.832 |
| ALT (u/L) | 24.1 ± 22.0 | 29.8 ± 29.1 | 0.094 |  | 26.8 ± 30.8 | 27.1 ± 20.2 | 0.877 |
| Total bilirubin (mg/dL) | 0.9 ± 0.7 | 0.9 ± 0.7 | 0.957 |  | 0.9 ± 0.6 | 0.9 ± 1.2 | 0.523 |
| LDL-C (mg/dL) | 91.6 ± 27.3 | 86.1 ± 25.7 | 0.436 |  | 94.9 ± 31.4 | 89.1 ± 27.2 | 0.168 |
| Medications |  |  |  |  |  |  |  |
| ß-blockers | 72 (58.1) | 57 (50.4) | 0.239 |  | 267 (59.2) | 185 (49.1) | 0.004 |
| RAAS inhibitors | 71 (57.3) | 65 (57.5) | 0.967 |  | 246 (54.6) | 229 (60.7) | 0.073 |
| Ca channel blockers | 31 (25.0) | 27 (23. 9) | 0.843 |  | 110 (24.4) | 109 (28.9) | 0.142 |
| Loop diuretics | 56 (45.2) | 61 (54.0) | 0.175 |  | 169 (37.5) | 199 (52.8) | <0.001 |
| Amiodarone | 45 (36.3) | 52 (46.0) | 0.128 |  | 154 (34.2) | 198 (52.5) | <0.001 |
| Digoxin | 25 (20.2) | 32 (28.3) | 0.142 |  | 71 (15.7) | 127 (33.7) | <0.001 |
| Statins | 35 (28.2) | 33 (29.2) | 0.868 |  | 141 (31.3) | 97 (25.7) | 0.080 |
| Aspirin | 46 (37.1) | 35 (31.0) | 0.321 |  | 154 (34.2) | 131 (34.8) | 0.856 |
| Clopidogrel | 30 (24.2) | 32 (28.3) | 0.471 |  | 110 (24.4) | 87 (23.1) | 0.659 |
| NSAIDs | 27 (21.8) | 29 (25.7) | 0.481 |  | 92 (20.4) | 70 (18.6) | 0.508 |
| PPIs | 91 (73.4) | 81 (71.7) | 0.769 |  | 290 (64.3) | 261 (69.2) | 0.134 |
| Steroid | 20 (16.1) | 19 (16.8) | 0.887 |  | 73 (16.2) | 59 (15.7) | 0.834 |

*eGFR, estimated glomerular filtration rate; DOACs, direct oral anticoagulants; NSAIDs, non-steroidal anti-inflammatory drugs; RAAS = renin–angiotensin–aldosterone system.*

**CHA_2_DS_2_-VASc score awards 1 point each for congestive heart failure, hypertension, diabetes mellitus, vascular disease, age 65–74 y, and female sex and 2 points each for age ≥75 y and previous stroke or transient ischemic attack.*

*^†^HAS-BLED score awards 1 point each for hypertension, abnormal renal or liver function, stroke, bleeding history, labile international normalized ratio, age ≥65 y, and antiplatelet drug or alcohol use.*

**Supplemental Table 5** Sensitivity analysis: DOAC versus warfarin in patients with PU with and without high bleeding risk

|  | **Rate (%/year)** | |  |  |
| --- | --- | --- | --- | --- |
|  | **DOACs** | **Warfarin** | **Crude HR (95% CI)** | ***p*-value** |
| PU with high rebleeding risk* (*n* = 157, DOAC: *n* = 85; warfarin: *n* = 72) | | | | |
| Major bleeding | 3.60 | 2.03 | 1.71 (0.16-18.86) | 0.662 |
| IS/SE | 13.08 | 4.19 | 2.99 (0.62-14.39) | 0.173 |
| Death | 7.20 | 4.04 | 1.66 (0.30-9.07) | 0.558 |
| Composite | 22.45 | 10.50 | 2.03 (0.72-5.77) | 0.183 |
| PU without high rebleeding risk^†^ (*n* = 908; DOAC: *n* = 490; warfarin: *n* = 418) | | | | |
| Major bleeding | 0.86 | 4.23 | 0.20 (0.06-0.71) | 0.013 |
| IS/SE | 2.88 | 2.92 | 0.96 (0.39-2.37) | 0.937 |
| Death | 5.13 | 5.10 | 0.97 (0.49-1.90) | 0.928 |
| Composite | 8.63 | 12.25 | 0.70 (0.43-1.13) | 0.140 |

*DOACs, direct oral anticoagulants; HR, hazard ratio; IS/SE, ischemic stroke/systemic embolism.*

**PU with high rebleeding risk was defined as Forrest Ia-IIb ulcer lesions according to the Forrest classification.*

*^†^PU without high rebleeding risk Inactive peptic ulcer was defined as Forrest IIc-III ulcer lesions according to the Forrest classification.*

**Supplemental Table 6** Sensitivity analysis: changing the study start date from January 1, 2009 to May 1, 2012.

|  | **Event rate /100 person-years** | |  | **Crude**  **HR (95% CI)** | ***p*-value** |  | **Adjusted**  **HR* (95% CI)** | ***p*-value** |  | **Competing risk^†^**  **HR* (95% CI)** | ***p*-value** |
| --- | --- | --- | --- | --- | --- | --- | --- | --- | --- | --- | --- |
|  | **DOACs** | **Warfarin** |  |  |  |  |  |  |  |  |  |
| Active PU | (*n* = 121) | (*n* = 75) |  |  |  |  |  |  |  |  |  |
| Major bleeding | 2.44 | 4.09 |  | 0.60 (0.08–4.23) | 0.604 |  | 0.93 (0.01–99.11) | 0.676 |  | 0.93 (0.10–8.74) | 0.949 |
| IS/SE | 10.00 | 4.16 |  | 2.41 (0.51–11.33) | 0.267 |  | 0.86 (0.16–4.74) | 0.865 |  | 0.67 (0.08–5.51) | 0.712 |
| Death | 8.52 | 12.06 |  | 0.71 (0.24–2.10) | 0.530 |  | 1.02 (0.31–3.38) | 0.972 |  |  |  |
| Composite | 18.76 | 21.19 |  | 0.89 (0.40–1.98) | 0.778 |  | 0.80 (0.34–1.92) | 0.623 |  |  |  |
| Inactive PU | (*n* = 448) | (*n* = 243) |  |  |  |  |  |  |  |  |  |
| Major bleeding | 0.94 | 4.98 |  | 0.19 (0.05–0.70) | 0.013 |  | 0.30 (0.07–1.29) | 0.107 |  | 0.46 (0.08–2.69) | 0.386 |
| IS/SE | 2.85 | 2.19 |  | 1.27 (0.39–4.14) | 0.687 |  | 1.48 (0.42–5.20) | 0.539 |  | 1.47 (0.42–5.10) | 0.544 |
| Death | 4.71 | 5.42 |  | 0.84 (0.38–1.86) | 0.659 |  | 0.64 (0.27–1.49) | 0.297 |  |  |  |
| Composite | 8.57 | 12.31 |  | 0.69 (0.39–1.20) | 0.188 |  | 0.72 (0.40–1.31) | 0.285 |  |  |  |
| No PU | (*n* = 1043) | (*n* = 555) |  |  |  |  |  |  |  |  |  |
| Major bleeding | 1.42 | 5.15 |  | 0.27 (0.13–0.57) | <0.001 |  | 0.26 (0.12–0.53) | <0.001 |  |  |  |
| IS/SE | 4.74 | 4.93 |  | 0.94 (0.54–1.64) | 0.822 |  | 0.92 (0.55–1.54) | 0.757 |  |  |  |
| Death | 4.65 | 4.33 |  | 1.04 (0.58–1.84) | 0.902 |  | 0.85 (0.50–1.45) | 0.558 |  |  |  |
| Composite | 10.52 | 13.31 |  | 0.77 (0.54–1.10) | 0.149 |  | 0.64 (0.45–0.93) | 0.019 |  |  |  |

*AF, atrial fibrillation; CI, confidence interval; DOAC, direct oral anticoagulant; HR, hazard ratio; IS/SE, ischemic stroke/systemic embolism; PU, peptic ulcer.*

*Active and inactive PU were defined as Forrest Ia-IIc and Forrest III ulcer lesions according to the Forrest classification, respectively.*

**Major bleeding adjusted for age, sex, hypertension, diabetes mellitus, chronic liver disease, history of heart failure, estimated glomerular filtration rate <60 mL/min/1.73m^2^, cancer, history of peptic ulcer disease, history of bleeding, history of stroke, nonsteroidal anti-inflammatory drug, proton pump inhibitors, and anti-platelets. IS/SE or death or composite, adjusted for age, sex, hypertension, diabetes mellitus, heart failure, estimated glomerular filtration rate <60 mL/min/1.73m^2^, cancer, prior stroke, prior myocardial infarction, and statins.*

*†Death was considered as a competing risk factor in the Cox model.*

**Supplemental Table 7** Rates and risk of outcomes in patients with peptic ulcer and NSAID use

|  | **Rate (%/year)** | | **Crude HR (95% CI)** | ***p*-value** | **Adjusted HR* (95% CI)** | ***p*-value** |
| --- | --- | --- | --- | --- | --- | --- |
|  | **DOACs** | **Warfarin** |  |  |  |  |
| NSAID use (*n* = 270; DOACs: *n* = 146, warfarin: *n* = 124) | | | | | | |
| Major bleeding | 2.63 | 3.08 | 0.85 (0.17–4.19) | 0.837 | 1.31 (0.22–7.65) | 0.766 |
| IS/SE | 3.56 | 4.13 | 0.83 (0.21–3.33) | 0.796 | 1.23 (0.24–6.47) | 0.803 |
| Death | 1.75 | 3.03 | 0.58 (0.10–3.45) | 0.546 | 0.76 (0.11–5.37) | 0.787 |
| Composite | 7.15 | 10.51 | 0.67 (0.26–1.70) | 0.398 | 0.89 (0.32–2.50) | 0.828 |
| No NSAID use (*n* = 795; DOACs: *n* = 429, warfarin: *n* = 366) | | | | | | |
| Major bleeding | 0.68 | 4.24 | 0.16 (0.04–0.73) | 0.018 | 0.21 (0.04–1.01) | 0.051 |
| IS/SE | 4.50 | 2.70 | 1.62 (0.65–4.07) | 0.302 | 1.58 (0.60–4.12) | 0.354 |
| Death | 6.84 | 5.69 | 1.15 (0.59–2.25) | 0.682 | 0.92 (0.45–1.86) | 0.807 |
| Composite | 11.76 | 12.57 | 0.92 (0.57–1.49) | 0.726 | 0.89 (0.54–1.48) | 0.648 |

*CI, confidence interval; DOACs, direct oral anticoagulants; HR,hazard ratio; IS/SE, ischemic stroke/systemic embolism; NSAID, nonsteroidal antiinflammatory drug.*

**Major bleeding, adjusted for age, sex, hypertension, diabetes mellitus, chronic liver disease, heart failure, estimated glomerular filtration rate <60 mL/min/1.73m^2^, bleeding history, cancer, proton pump inhibitors, and steroid.*

**Supplemental Table 8** Event rate and risk of outcomes in AF patients with and without Helicobacter Pylori infection detected by histological examinations

|  | **Rate (%/year)** | |  |  | |
| --- | --- | --- | --- | --- | --- |
|  | **DOACs** | **Warfarin** |  | **Crude HR (95% CI)** | **p-value** |
| Helicobacter pylori infection (*n* = 90; DOACs: *n* = 55, warfarin: *n* = 35) | | | | | |
| Major bleeding | 0 | 6.70 |  | 0 (0–0) | <0.001 |
| IS/SE | 6.67 | 4.36 |  | 1.42 (0.28–7.31) | 0.673 |
| Death | 6.44 | 4.20 |  | 1.41 (0.29–6.97) | 0.670 |
| No Helicobacter pylori infection (*n* = 484; DOAC: *n* = 274, warfarin: *n* = 210) | | | | | |
| Major bleeding | 2.12 | 3.36 |  | 0.61 (0.21–1.80) | 0.370 |
| IS/SE | 2.13 | 2.08 |  | 1.01 (0.31–3.28) | 0.988 |
| Death | 6.65 | 7.83 |  | 0.79 (0.42–1.48) | 0.462 |

*AF, atrial fibrillation; CI, confidence interval; DOACs, direct oral anticoagulants; HR, hazard ratio; IS/SE, ischemic stroke/systemic embolism.*
